# MHC-I–Driven Antitumor Immunity Counterbalances Low Absorbed Doses of Radiopharmaceutical Therapy

**DOI:** 10.2967/jnumed.124.268857

**Published:** 2025-05

**Authors:** Julie Constanzo, Aliasghar Parach, Timothee David, Joshua Karam, Frank Bruchertseifer, Alfred Morgenstern, Marta Jarlier, Manuel Bardiès, Emmanuel Deshayes, Amelie Gudin-de-Vallerin, Florence Boissière-Michot, Evelyne Lopez-Crapez, Jean-Pierre Pouget

**Affiliations:** 1 Institut de Recherche en Cancérologie de Montpellier (IRCM), INSERM U1194, Université de Montpellier, Nuclear Medicine Department, Institut régional du Cancer de Montpellier (ICM), Montpellier, France, and Équipe Labellisée Ligue Contre le Cancer, Paris, France;; 2 Joint Research Centre, European Commission, Karlsruhe, Germany;; 3 Biometrics Unit, Institut Régional du Cancer Montpellier, Montpellier, France; and; 4 Translational Research Unit, Institut Régional du Cancer Montpellier, Montpellier, France

**Keywords:** radioimmunotherapy, targeted radionuclide therapy, ^225^Ac, microenvironment, bystander immunity, T-cell adaptive immunity

## Abstract

Preclinical and clinical studies increasingly show that the immune response plays a major role in radiotherapy. Here, we investigated the role of major histocompatibility complex class I (MHC-I) molecules recognized by cytotoxic CD8^+^ T cells in the response to radiopharmaceutical therapy (RPT). **Methods:** Two murine melanoma cell lines that express low and high MHC-I levels (B16F10 and B16K1, respectively) were grafted in syngeneic or athymic and nude mice, and the response to a single injection of [^225^Ac]Ac-DOTA-TA99 monoclonal antibodies (9.25 or 18.5 kBq) was assessed and related to dosimetry. For clinical relevance, MHC-I expression was determined in samples from patients with well-differentiated, iodine-avid metastatic thyroid cancer and well-differentiated grade 2 mid-gut neuroendocrine tumors. **Results:** RPT efficacy was enhanced by T-cell presence and MHC-I expression. In mice harboring B16F10 and B16K1 melanoma tumors, RPT showed a stronger antitumor effect in C57BL/6J (immunocompetent) animals than in athymic and nude (immunodeficient) animals, suggesting a crucial role of T-cell–mediated immune responses. Moreover, the response to irradiation was the same in B16K1 MHC-I_high_ tumors with a low absorbed dose of α-RPT and in B16F10 MHC-I_low_ tumors with a 4 times higher absorbed dose. These results indicate that CD8^+^ T cells can counterbalance low tumor irradiation. Conversely, delivering high absorbed doses leads to side effects and seems to prevent immune system activation, thereby not taking advantage of these mechanisms. Our results also indicate that MHC-I can be used as a predictive biomarker of RPT response in lesions receiving low absorbed doses and that RPT treatment regimens should be reconsidered in the function of the MHC-I expression level. **Conclusion:** This study shows that MHC-I expression can predict RPT immunostimulatory effects. This is relevant in metastatic disease where lesions in the same patient can receive very low or very high absorbed doses.

## Visual Abstract

**Figure:**
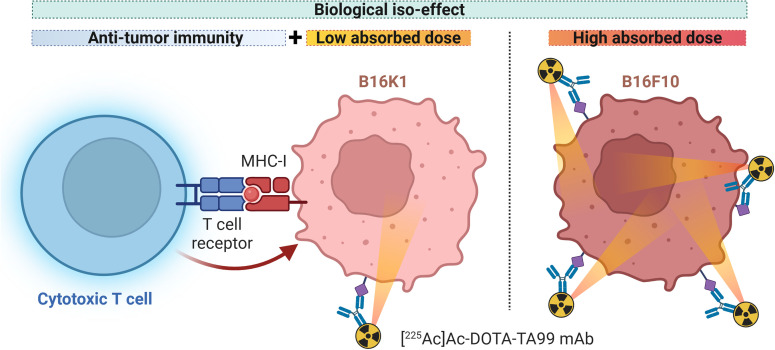

Tumor absorbed dose and tumor volume are among the main parameters involved in the assessment of external beam radiation therapy (EBRT) efficacy (1). In contrast, although absorbed dose or volume changes are considered in radiopharmaceutical therapy (RPT), they are not considered at the therapy planning stage (2). Major RPT features are the range of lesion absorbed doses among patients but also in the same patient (e.g., 10–300 Gy for neuroendocrine tumor lesions treated with [^177^Lu]Lu-DOTATATE) (3), the low absorbed dose rate (∼0.1 Gy/h), and lesion absorbed dose heterogeneity because of diverse ligand distribution and a limited particle range (4–6). These 2 last features favor intercellular communication between tumor cells (bystander cytotoxicity) (7*,*^8^) and between irradiated tumor cells and immune cells (bystander immunity) (9–11). Consequently, the tumor response to RPT is influenced not only by the absorbed dose but also by the patient and tumor biologic parameters, including the immune response and tumor microenvironment (11–13). Several preclinical studies and a few clinical studies have indicated that immune response contributes to RPT efficacy. RPT can induce immunogenic cell death by triggering an antitumor immune response through the increase of adjuvanticity and antigenicity. Major histocompatibility complex class I (MHC-I) is one of the immune response actors. MHC-I is a heterodimer of 2 domains: a polymorphic human leukocyte antigen-encoded heavy α-chain and an invariant light chain called β_2_-microglobulin. MHC-I molecules are expressed at the (tumor) cell surface (14). After binding to cytoplasmic antigenic peptides that result from protein hydrolysis, MHC-I molecules are translocated into the endoplasmic reticulum, where peptide MHC-I is formed and then translocated to the cell membrane through Golgi vesicles. Then, peptide MHC-I can be displayed to cytotoxic CD8^+^ T cells. Carretero et al. (15) observed after autologous vaccination of patients with subcutaneous melanoma lesions a massive intratumor infiltration of CD4^+^ and CD8^+^ T lymphocytes in regressing melanoma lesions that was associated with high MHC-I levels. Conversely, progressing lesions were associated with the absence of tumor-infiltrating lymphocytes, low MHC-I levels, and mostly peritumoral infiltration patterns (15). MHC-I loss and reduced expression are associated with worse overall survival and with intrinsic and acquired resistance to immunotherapy, including immune checkpoint inhibitors (16–18). Moreover, in some cancer cell lines, EBRT can increase the cell surface expression of MHC-I molecules for many days in an absorbed dose–dependent manner (19–21). In addition, conditioned medium from irradiated cells (3 × 2 Gy) can increase MHC-I expression in radiation-naïve recipient cells (22). These results demonstrate that radiation can induce MHC-I expression directly or indirectly through bystander signaling, thereby enhancing the antitumor immune response. Therefore, this study wanted to determine whether MHC-I expression plays a role in RPT and how this information can be used to improve RPT clinical therapeutic efficacy.

## MATERIALS AND METHODS

### Cell Lines

The murine melanoma cell lines B16F10 (purchased from the American Type Culture Collection) and B16K1, genetically modified from B16F10 cells to stably express the MHC-I molecule H-2Kb (23), were grown in RPMI 1640 medium supplemented with 10% fetal bovine serum and 1% penicillin (i.e., complete medium).

### Animal Models

Animal models are described in the supplemental materials (supplemental materials are available at http://jnm.snmjournals.org).

### TA99 Antibody Radiolabeling and Treatment

The antimouse tyrosinase-related protein 1 (TYRP-1, also known as TRP-1) TA99 monoclonal antibody (mAb; IgG2a format) was purchased from BioXcell. TYRP-1/gp75 is expressed on the surface and intracellularly in human and mouse melanoma cells (24). At day 5 after grafting, mice were randomized (*n* = 7–8/group) to receive a single intraperitoneal injection (200 µL) of 0.9% sodium chloride, low activity of [^225^Ac]Ac-DOTA-TA99 (9.25 kBq; 0.8 nmol; molar activity, 11.6 kBq/nmol; in 100 µL of 0.9% sodium chloride), or medium activity of [^225^Ac]Ac-DOTA-TA99 (18.5 kBq; 0.8 nmol; molar activity, 23.1 kBq/nmol; in 100 µL of 0.9% sodium chloride). ^225^Ac, received as a nitrate salt, was reconstituted with 0.2 M Optima-grade (Fisher Scientific) hydrochloric acid solution (10 µL/37 MBq, pH 1). A solution of TA99-DOTA (4.82 mg/mL; DOTA/mAb ratio, 2 ± 1) in 0.25 M ammonium acetate buffer (pH 7) was prepared. Then, the desired activity (9.25 or 18.5 kBq) was added to the reaction mixture (for a specific activity of 37 kBq/µg), and the solution was incubated at 40°C for 1 h. After purification on PD-10 desalting columns, instant thin-layer chromatography was used to assess the presence of free nonequilibrium daughters at a low level (<5%). At secular equilibrium, radioligands were obtained with radiochemical purity of more than 90%.

### Clonogenic Cell Survival

The radiation sensitivity of the 2 cell lines to [^225^Ac]Ac-DOTA-TA99 (9.25 and 18.5 kBq/mL) was assessed using a standard clonogenic assay, as previously described (25*,*^26^).

### Hematotoxicity

Hematotoxicity monitoring is described in the supplemental materials.

### Biodistribution and Dosimetry

The TA99 mAb biodistribution, using [^111^In]In-DTPA-TA99 mAb (DTPA/mAb ratio, 1 ± 1) as surrogate (27), was evaluated in healthy organs or tissues and tumors collected from B16F10 and B16K1 cell-grafted mice using a γ-counter, as previously described (28). The dosimetry procedures are described in the supplemental materials.

### Flow Cytometry Analysis

Cells were incubated with [^225^Ac]Ac-DOTA-TA99 (9.25 and 18.5 kBq/mL) for 90 min or exposed to EBRT (2 and 8 Gy) using 225-kV x-rays at a 2 Gy/min absorbed dose rate (XenX; Xstrahl). EBRT absorbed doses were chosen based on their capacity to increase MHC-I expression (19*,*^29^*,*^30^). After incubation, cells were washed and fresh medium was added; untreated cells were used as negative controls, and recombinant interferon-γ (IFN-γ) was used as positive control. Cells were harvested at 24 and 48 h after treatment, washed, and stained with Viakrome 808 viability dye (Beckman Coulter) and a phycoerythrin-conjugated anti-MHC-I antibody. After additional washes, cells were fixed and analyzed using a CytoFLEX LX flow cytometer (Beckman Coulter). Data analysis excluded debris, gated live cells, and extracted phycoerythrin-channel geometric mean fluorescence intensity using FlowJo version 10.10 (FlowJo LLC). Flow cytometry procedures are fully described in the supplemental materials.

### Human Samples

Human specimens were from 7 patients (7 samples) with iodine-avid, papillary thyroid carcinoma treated with 3.7 GBq of [^131^I]I and 5 patients (5 samples) with well-differentiated grade 1 or 2 metastatic, mid-gut neuroendocrine tumors treated with 7.4 GBq of [^177^Lu]Lu-DOTATATE (≤4 cycles) upfront or after 1 treatment line with somatostatin analogs (Table 1). All patients signed an informed-consent form. Samples were identified from the clinical-biologic BCB RIV database (NCT04104529) and managed by the Montpellier Cancer Institute Biologic Resource Center (tumor biobank number BB-0033-00059). The study was approved by the institutional ethics review board (ICM-CORT 2024/09). Tumor samples before RPT from these 2 patient groups were used to assess the baseline MHC-I expression level.

**TABLE 1.** Characteristics of Patients and Tumors

Patient no.	Sex	Age at surgery (y)	Histologic type	Localization	Grade	Stage	MHC-I Q score	CD8^+^ T-cell infiltrate
1	M	72	Papillary carcinoma, classic subtype	Thyroid	NA	pT2NxM+x	10	+
2	M	65	Papillary carcinoma, vesicular variant subtype	Thyroid	NA	pT4aN1bM+x	200	+++
3	M	53	Papillary carcinoma, classic subtype	Thyroid	NA	pT3bN+0M0x	170	++
4	F	48	Papillary carcinoma, classic subtype	Thyroid	NA	pT1bN1bM0x	190	++
5	F	53	Papillary carcinoma, diffuse sclerosing subtype	Thyroid	NA	pT1(m)N1aM0x	300	+++
6	F	51	Papillary carcinoma, vesicular variant subtype	Thyroid	NA	pT3(m)NxM+x	0	+
7	M	53	Papillary carcinoma, vesicular variant subtype	Thyroid	NA	pT3aNxM+x	2	+
8	M	63	NET	Small intestine	2	pT4N1M1x	1	+
9	M	59	NET	Ileum	2	pT4N2M1x	80	++
10	F	73	NET	Small intestine	2	pT4N1M1	10	+
11	F	73	NET	Ileum	2	pT3N1M1	5	+
12	F	58	NET	Lymph node metastasis of NET from small intestine	1	pT3N1M1	5	+

NA = not applicable; + = weak; ++ = moderate; +++ = strong; NET = neuroendocrine tumor.

### Immunohistochemical Procedures

Serial sections (3 µm thick) were obtained from formalin-fixed paraffin-embedded tumor blocks. One section was stained with hematoxylin–eosin–saffron, and the other 2 were used for MHC-I and CD8 detection. Only membrane MHC-I expression was considered positive. MHC-I Q scores were obtained by multiplying the staining intensity (0, no staining; 1, weak staining; 2, moderate staining; 3, intense staining) by the percentage of MHC-I–positive cells. This score produced a continuous variable that ranged from 0 to 300 (Table 1). CD8^+^ T-cell infiltration was subjectively quantified as weak, moderate or strong, according to the amount of CD8^+^ T cells in the tumor parenchyma (Table 1). All immunohistochemical procedures are described in the supplemental materials.

### Statistical Analysis

All statistical analysis procedures are described in the supplemental materials.

## RESULTS

### B16K1 Melanoma Cells Express MHC-I Strongly and TYRP-1 Weakly

**FIGURE 1.**
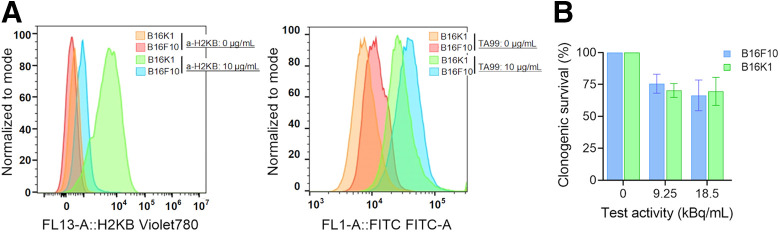
Cell line characterization. (A) Flow cytometry analysis of MHC-I (anti–H-2Kb antibody) and TYRP-1/gp75 (TA99 antibody) expression in B16F10 and B16K1 melanoma cells. (B) Radiation sensitivity of 2 cell lines. FITC = fluorescein isothiocyanate.

To evaluate MHC-I role in the immune response during RPT, we selected the B16F10 and B16K1 melanoma cell lines that constitutively express MHC-I (H-2Kb) at low and high levels, respectively (Fig. 1A). On the basis of Shklovskaya et al. (31), the B16F10 and B16K1 models are representative of the MHC-I expression heterogeneity observed in patients with melanoma. Conversely, the TYRP-1 expression level was higher in B16F10 than in B16K1 cells (Fig. 1A). In vitro, the difference in TYRP-1 receptor expression between the 2 cell lines does not imply a significant difference in intrinsic radiosensitivity that may have arisen after genetic modification (B16K1 cells), as indicated by the clonogenic survival assay results (Fig. 1B).

### RPT Efficacy Is Positively Influenced by T-Cell Presence and MHC-I Expression

**FIGURE 2.**
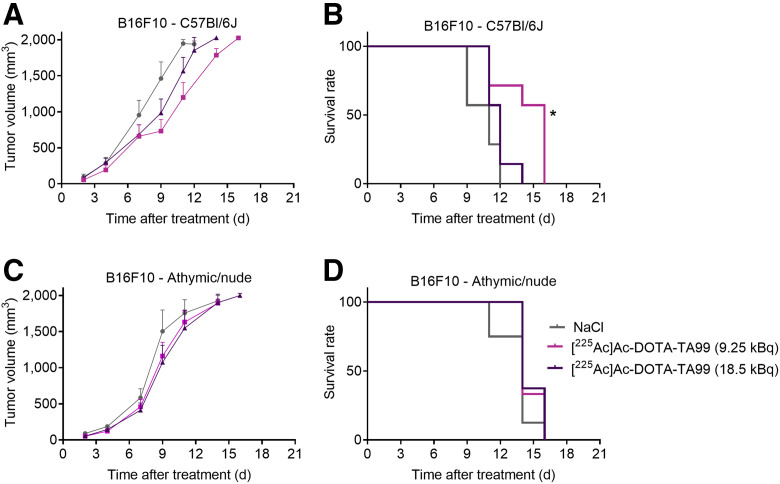
In vivo therapeutic efficacy of [^225^Ac]Ac-DOTA-TA99 mAb (9.25 and 18.5 kBq) in B16F10 cell–bearing C57BL/6J mice (A and B) and athymic and nude mice (C and D). Results are mean ± SEM. *P < 0.05, **P < 0.01, based on log-rank test. NaCl = sodium chloride.

Next, we investigated the therapeutic efficacy of a single administration of [^225^Ac]Ac-DOTA-TA99 (9.25 and 18 kBq) in immunocompetent (C57BL/6J) and immunodeficient (athymic and nude) mice subcutaneously grafted with B16F10 and B16K1 melanoma cells. Compared with sodium chloride (control group), the RPT antitumor effect was higher in immunocompetent C57BL/6J mice than in athymic and nude mice harboring B16F10 tumors (Fig. 2), suggesting a role for T-cell–mediated adaptive immune response. The median survival (MS) was 14 d in C57BL/6J mice treated with 9.25 kBq of [^225^Ac]Ac-DOTA-TA99 (vs. 11 d in the control group; *P* = 0.045) and 11 d in athymic and nude mice treated with 9.25 kBq of [^225^Ac]Ac-DOTA-TA99 (vs. 11 d for the control group), based on a tumor volume of 1,800 mm^3^ (Figs. 2B–2D). When activity was increased to 18.5 kBq, the MS decreased (to 12 d) in C57BL/6J mice but remained unchanged and similar to that of the control group in athymic and nude mice (11 d).

**FIGURE 3.**
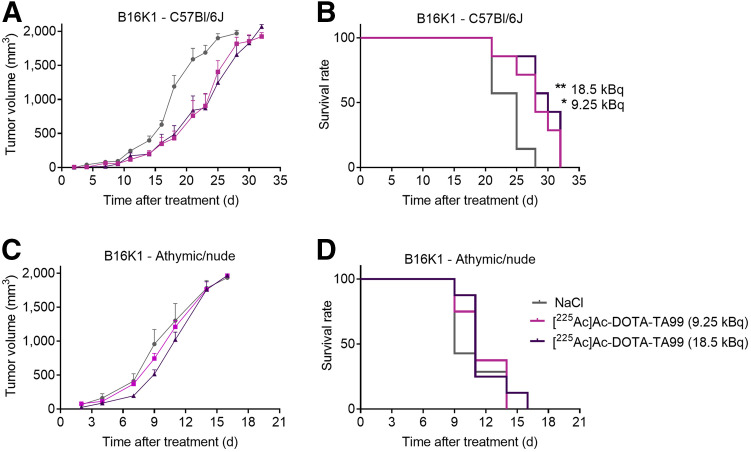
In vivo therapeutic efficacy of [^225^Ac]Ac-DOTA-TA99 mAb in B16K1 cell–bearing C57BL/6J mice (A and B) and athymic and nude mice (C and D). Results are mean ± SEM. *P < 0.05, **P < 0.01, based on log-rank test. NaCl = sodium chloride.

We obtained similar results with the B16K1 melanoma model. RPT efficacy was higher in C57BL/6J mice (MS, 28 d at 9.25 kBq and 30 d at 18.5 kBq vs. 25 d for the control group; *P* = 0.026 and *P* = 0.009, respectively; Figs. 3A and 3B) than in athymic and nude mice, in which RPT was ineffective at both 9.25 and 18.5 kBq of [^225^Ac]Ac-DOTA-TA99 (Figs. 3C and 3D). These findings indicated that the MS after RPT was higher in the B16K1 than in the B16F10 melanoma model. B16F10 cells grew faster in vivo than did B16K1 cells, possibly because of their lower MHC-I expression level (different immunogenicity).

### High α-RPT Activity Induces Bone Marrow Toxicity

**FIGURE 4.**
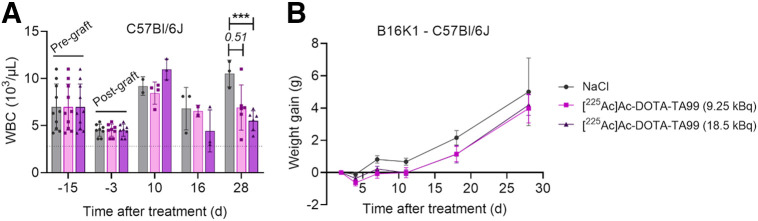
Toxicity of [^225^Ac]Ac-DOTA-TA99 mAb in immunocompetent mice bearing B16K1 melanoma cell grafts. (A) White blood cell count. (B) Weight measurement in mice after RPT. Results are mean ± SEM. *P < 0.05. **P < 0.01. ***P = 0.0005. NaCl = sodium chloride.

Because MS was lower in B16F10 melanoma–harboring mice treated with 18.5 kBq than in those treated with 9.25 kBq of [^225^Ac]Ac-DOTA-TA99, we asked whether bone marrow toxicity and the resulting white blood cell decrease could explain this finding. Our hypothesis was that high activities do not counterbalance the loss of immune cells due to bone marrow toxicity. Because bone marrow toxicity is generally observed around 14 d after RPT in mice, we could assess it only in B16K1 cell–harboring C57BL/6J mice that showed longer survival. At day 28 after RPT, the white blood cell count was significantly decreased (vs. the control group) in mice that received 18.5 kBq (*P* = 0.0005) but not significantly decreased in mice that received 9.25 kBq of [^225^Ac]Ac-DOTA-TA99 (Fig. 4A). Moreover, weight loss of less than 5% of the total body weight was observed at day 4 after treatment (both activities), but weight was fully recovered at day 7 after RPT (Fig. 4B).

### Absorbed Doses Are Lower in B16K1 than in B16F10 Tumors

**FIGURE 5.**
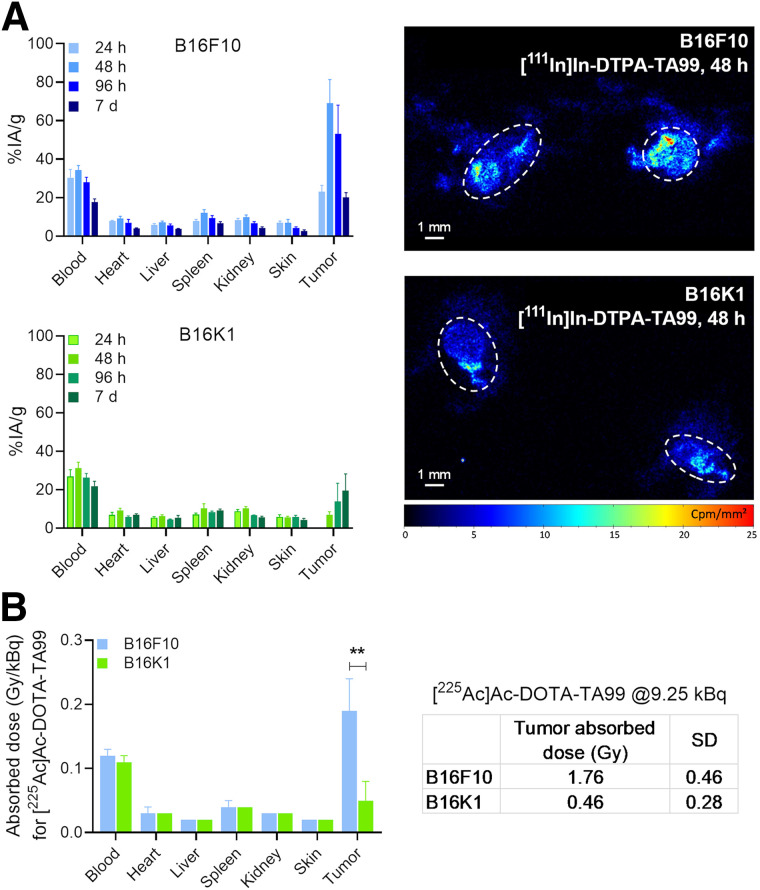
Biodistribution and dosimetry. (A) Biodistribution of [^111^In]In-DTPA-TA99 mAb was determined by ex vivo γ-radiation counting of tumor nodules and organs collected at various times (*n* = 3–4 per time point) after intraperitoneal injection in mice harboring B16F10 and B16K1 tumors (left), with corresponding digital autoradiography images of tumors (delineated by circles) at 48 h after injection (right). (B) Absorbed dose in Gy/kBq was determined for each organ (left), and tumor absorbed dose for [^225^Ac]Ac-DOTA-TA99 (9.25 kBq) was assessed, with SD (right). Histograms are mean ± SEM. *P < 0.05, based on 2-tailed parametric unpaired *t* test. **P < 0.01, based on 2-tailed parametric unpaired *t* test. %IA/g = percentage injected activity per gram of tissue.

The absorbed dose is 1 key parameter involved in RPT response. Therefore, we determined the biodistribution of the TA99 mAb, using [^111^In]In-DTPA-TA99 mAb, in both tumor models. The maximum uptake at 48 h was 69.1 ± 12.3 and 7.1 ± 1.5 percentage injected activity per gram of tissue in B16F10 and B16K1 tumors, respectively (Fig. 5A, left). Digital autoradiography showed that the radioligand spatial biodistribution was similar in the 2 tumor models (Fig. 5A, right). Then, we calculated the tumor and normal tissue absorbed doses from [^111^In]In-DTPA-TA99 pharmacokinetics extrapolated to [^225^Ac]Ac-DOTA-TA99 by considering differences in specific activity and physical half-life. The blood absorbed doses were similar in the 2 tumor models: 0.12 ± 0.01 Gy/kBq for B16F10 and 0.11 ± 0.01 Gy/kBq for B16K1 (Fig. 5B, left). Conversely, the tumor absorbed dose was significantly higher in B16F10 tumors (0.19 ± 0.05 Gy/kBq) than in B16K1 tumors (0.05 ± 0.03 Gy/kBq; *P* = 0.001). When applied to the in vivo experimental conditions of therapeutic studies (Figs. 2 and 3), we obtained tumor absorbed doses of 1.76 ± 0.5 and 0.46 ± 0.28 Gy in B16F10 and B16K1 cell grafts, respectively, after treatment with 9.25 kBq of [^225^Ac]Ac-DOTA-TA99.

### RPT Upregulates MHC-I Expression Only in Tumor Cells That Display High Basal MHC-I Expression

**FIGURE 6.**
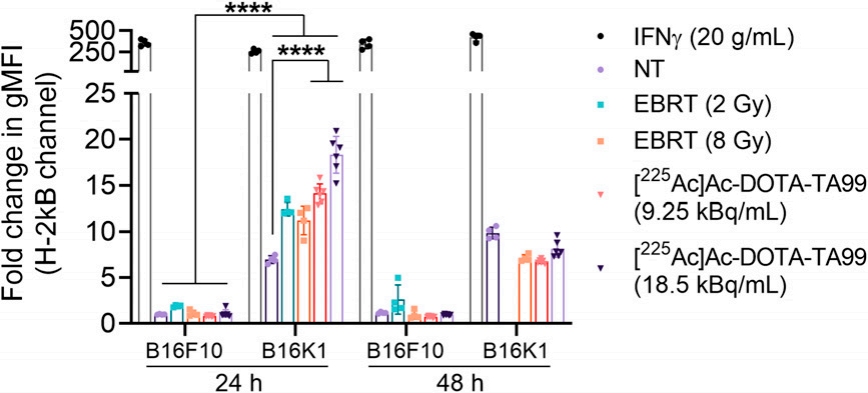
Modulation of MHC-I (H-2Kb) expression on EBRT and RPT. Results are mean ± SD. ****P < 0.0001, based on 2-tailed parametric unpaired *t* test. IFN-γ is positive control. gMFI = geometric mean fluorescence intensity; NT = not treated.

We next assessed in vitro MHC-I expression modulation by RPT and EBRT in B16F10 and B16K1 cells. Neither EBRT (2 and 8 Gy) nor RPT (9.25 and 18.5 kBq) increased MHC-I expression in B16F10 cells during the 48 h after exposure. Conversely, the MHC-I level was transiently increased in B16K1 cells at 24 h after exposure. Specifically, MHC-I geometric mean fluorescence intensity was increased by about 12-fold after exposure to EBRT (2 and 8 Gy), by 15-fold after exposure to 9.25 kBq of [^225^Ac]Ac-DOTA-TA99 as RPT (*P* = 0.025 vs. 2-Gy EBRT), and by about 20-fold after exposure to 18.5 kBq of [^225^Ac]Ac-DOTA-TA99 (*P* = 0.001 vs. 9.25 kBq; Fig. 6). Only IFN-γ (positive control) increased MHC-I level (250-fold change in geometric mean fluorescence intensity) in both melanoma models. These results indicate that MHC-I expression is upregulated by RPT or EBRT only in tumor cells that already display a high MHC-I level.

### High MHC-I Expression Is Associated with High CD8^+^ T-Cell Density in Patients’ Tumor Samples Before RPT

**FIGURE 7.**
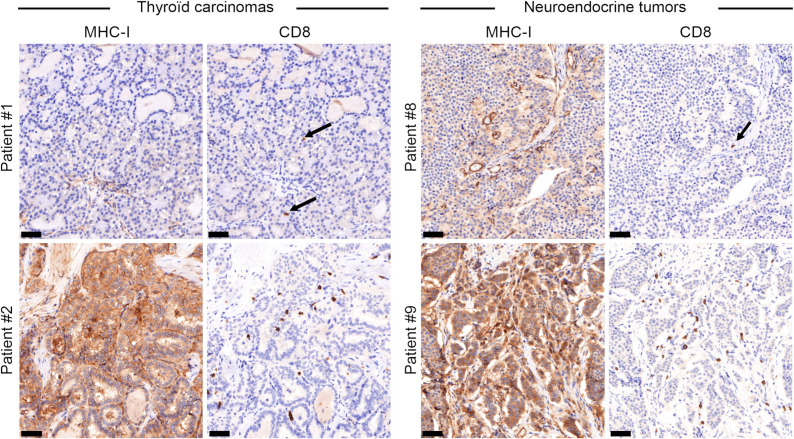
MHC-I expression and CD8^+^ T-cell density in thyroid carcinoma and neuroendocrine tumor samples. Variable patterns of MHC-I immunoreactivity and CD8^+^ T-cell density are seen. In both tumor types, low MHC-I expression level (patients 1 and 8) was associated with low CD8^+^ T-cell density (arrows), whereas high MHC-I expression level (patients 2 and 9) was associated with higher CD8 ^+^ T-cell density. Scale bars are 50 µm.

Lastly, we investigated MHC-I expression and CD8^+^ T-cell density in thyroid carcinoma (*n* = 7) and neuroendocrine tumor (*n* = 5) samples from patients before ([^131^I]I or [^177^Lu]Lu-DOTATATE) RPT to determine whether these patients could be good candidates for RPT-mediated MHC-I upregulation, considering that MHC-I could counterbalance the low absorbed doses delivered to some lesions. We observed different MHC-I immunoreactivity levels and CD8^+^ T-cell density profiles in samples; however, in both tumor types, a low MHC-I expression level was associated with low CD8^+^ T-cell density and a high MHC-I expression was associated with higher CD8^+^ T-cell density (Table 1; Fig. 7; Supplemental Figs. 1 and 2).

## DISCUSSION

There is a growing enthusiasm for α-RPT, largely driven by promising initial clinical outcomes observed in a limited number of patients, along with radiobiologic data that, although encouraging, are predominantly derived from studies using immunocompromised models or in vitro systems. However, there remains a significant knowledge gap in our understanding of α-RPT’s mechanisms and effects. Although it has been established that an α-particle is 4–5 times more cytotoxic than a β-particle (32), the influences of the absorbed dose, the absorbed dose rate, and the radiation quality on therapeutic outcomes remain poorly understood. This is particularly concerning in a context in which particles that are more cytotoxic to tumor cells also exhibit increased toxicity to healthy tissues (off-target effects), especially radiosensitive organs such as bone marrow. Furthermore, although DNA double-strand breaks (including cytosolic DNA) are known to trigger an antitumor immune response (33*,*^34^), the formation and implications of such lesions in the context of α-RPT remain largely unexplored. Here, melanoma cell grafts that strongly express MHC-I molecules responded better to α-RPT than did tumors with low MHC-I expression that received absorbed doses that were 4 times higher than those received by high MHC-I tumors. Moreover, RPT response was higher in immunocompetent than in immunodeficient mice, confirming the T-cell role when low activities (9.25 kBq) of [^225^Ac]Ac-DOTA-TA99 are used. This effect was not observed when higher activities (18.5 kBq) of [^225^Ac]Ac-DOTA-TA99 were used. Bone marrow toxicity occurrence supports the hypothesis that high activities hinder the immune system activation. In addition, RPT increased the MHC-I expression level only in melanoma cells that already strongly expressed MHC-I molecules. Lastly, although MHC-I immunoreactivity and CD8^+^ T-cell density varied in patients with thyroid carcinoma and mid-gut neuroendocrine tumors, overall, high MHC-I expression was associated with higher CD8^+^ T-cell density, which is a favorable prognostic factor. Altogether, these data indicate that MHC-I–mediated antigen presentation is critical for CD8^+^ T-cell responses and plays a key role in the RPT-induced adaptive immune response.

We think that these observations are highly relevant for RPT in which the lesion absorbed doses are strongly heterogeneous among patients and in the same patient. Hebert et al. (3) demonstrated that in patients receiving the same treatment (7.4 GBq of [^177^Lu]Lu-DOTATATE per cycle for 4 cycles), some lesions received 20 Gy and others received up to 300 Gy. Strikingly, the authors did not observe complete lesion regression even at the highest absorbed dose (300 Gy). If tumor lesions express high basal levels of MHC-I, we could expect all lesions, even those receiving low absorbed doses, to respond similarly to RPT. Moreover, increasing the absorbed dose is questionable, because it induces side effects and may hinder the CD8^+^ T-cell role in RPT efficacy due to bone marrow and spleen toxicity (35*,*^36^). A fine balance needs to be found for more personalized RPT.

The surrogate agent [^111^In]In-DTPA-TA99 mAb was used for dosimetry in this study, which aimed to compare the efficacy of 2 different activities in 2 tumor models. Differences in organ distribution, particularly in kidneys and liver, can arise due to variations in the clearance and catabolism of the chelates ([^225^Ac]Ac-DOTA vs. [^111^In]In-DTPA) in the antibody conjugate (3). In addition, our dosimetry calculation assumed that actinium and its decay products are located at the same site and at the same time as indium. Although these parameters may influence dosimetry calculations, the observed factor of 4 in absorbed dose difference between B16F10 and B16K1 tumors should remain a relevant index of the relative irradiation delivered by the 2 radioimmunoconjugates.

These data drastically change our view on RPT efficacy and radiobiology. Because high absorbed doses and a high absorbed dose rate are usually sought in EBRT, the continuous low absorbed dose rate of RPT could be seen as a disadvantage. However, our results showing the lower efficacy of 18.5 versus 9.25 kBq of [^225^Ac]Ac-DOTA-TA99 suggested that this paradigm cannot be directly extrapolated to RPT (32). Because the maximal tolerated activity of ^225^Ac-labeled mAb is more than 37 kBq, the 2 activities used in this study are relatively low. The role of irradiation on immune system activation has already been described in several in vitro and in vivo studies using EBRT and RPT. In vitro, the antitumor immune response is triggered at absorbed doses ranging from 8 to 12 Gy (33). In vivo, in syngeneic mice bearing mammary carcinoma cells at 2 separate sites, fractionated irradiation (e.g., 6 Gy in 5 fractions) of the primary tumor site led to regression of the distant, nonirradiated tumor site. This effect was not observed with an absorbed dose of 20 Gy (37). Unlike the usual RPT studies at tumor cell–destroying absorbed doses (or maximal tolerated activity), Patel et al. (9) evaluated RPT immunomodulatory effects at low absorbed doses (2.5 Gy; ^90^Y-labeled alkyl phosphocholine, NM600) and 2.5 Gy of EBRT. Innate myeloid (CD11b^+^) and natural killer cells were significantly higher at day 7 after low RPT irradiation. Moreover, the ratio of effector cells (CD8^+^) to suppressor regulatory T cells (CD4^+^CD25^+^FOXP3^+^) was increased in the RPT group, at day 1 after treatment, from that of the EBRT group, although the absorbed dose was equivalent (9). This study shows that RPT with a low absorbed dose can reprogram the tumor microenvironment and convert cold tumors into immunoreactive and immune checkpoint blockade-responsive tumors. Moreover, recent preclinical and clinical studies suggest that EBRT with a low absorbed dose can effectively mobilize innate and adaptive immunity (38–41). Herrera et al. (39*,*^40^) studied the effect of low-absorbed-dose EBRT in a syngeneic ID8 ovarian cancer mouse model in which T cells are fully exhausted, as observed in many human epithelial ovarian carcinomas that are naturally resistant to immune checkpoint inhibitors. In a phase 1 study, they found that whole-abdomen EBRT (0.5–2 Gy) in patients could transform cold tumors into hot tumors by increasing the density of lymphocytes, monocytes, dendritic cells, and natural killer cells in the tumor microenvironment. Lejeune et al. (10) demonstrated that in preclinical tumor models, α-RPT induces transcriptional and molecular signatures of immunogenic cell death, culminating in the activation of a therapeutically relevant tumor-targeting immune response, which can be amplified by immune checkpoint inhibitors. These findings align with those observed in β-RPT (42).

Our study suggests that a threshold MHC-I expression level is required to observe its increase on RPT and EBRT (Fig. 6). As in B16F10 cells (MHC-I^low^), Boreel et al. (43) did not detect in vivo any MHC-I signal increase in MOC1.3D5 (MHC-I^low^) tumor cells (syngeneic murine model) at days 3 and 10 after treatment, regardless of EBRT absorbed doses (0, 6, 12, and 18 Gy). Conversely, in EBRT, an absorbed dose threshold (19) and MHC-I expression kinetics may exist, as evidenced by the increase in MHC-I expression in CT26 cells at 24 h after irradiation in vitro (10 Gy) (29). Moreover, [^211^At]At-succinimidyl astatobenzoate-octreotide (targeting somatostatin receptor 2–positive small cell lung cancer) and [^223^Ra]RaCl_2_ (targeting human carcinoma cells) upregulated MHC-I expression on the tumor cell membrane surface (44*,*^45^).

In addition, the MHC-I expression level is variable within the same tumor type (Fig. 7). This suggests that tumors with a sufficiently high MHC-I expression level might respond to low absorbed doses of RPT by counterbalancing the lack of irradiation through immune system activation. We previously suggested not only that the absorbed dose delivered to lesions needs to be above a threshold (3*,*^5^) but also that very high absorbed doses might be more toxic than beneficial. Several proof-of-concept studies using ovalbumin-expressing cancer models showed that radiation can induce upregulation of tumor-specific antigens. In vitro, Lai et al. (46) showed that in MC38-ovalbumin and EG7-ovalbumin tumor cells, radiation significantly increase MHC-I expression, as seen in the present study. They also demonstrated that the ovalbumin-derived SIINFEKL peptide is presented more strongly by MHC-I (H-2Kb) after irradiation. On irradiation of mice grafted with AE17-ovalbumin (47), or B16F10-ovalbumin and 4T1-hemagglutinin (48) cancer cells, MHC-I or antigen-specific peptide complexes were increased, followed by anti-CD8^+^ T-cell–mediated antitumor response priming. Tailor et al. (29) analyzed the immunopeptidome of irradiated CT26 cells and found that radiation induced specific antigens, including peptides associated with catecholamine signaling. On the basis of these findings and our results, we suggest that radiation enhances MHC-I presentation of radiation- and mainly tumor-specific peptide antigens after EBRT. Conversely, it remains undetermined for RPT.

In patient tumors before RPT, we found an association between high MHC-I expression and increased CD8^+^ T-cell presence. However, the relationship between immunoscoring (49) at diagnosis and the onset of RPT (which may occur months or years later) could differ. Furthermore, the impact of RPT on MHC-I expression after 1 or more treatment cycles remains unclear. Monitoring changes in these markers after multiple RPT cycles would provide insights into the RPT-induced antitumor immunity. In addition, biopsy is rarely performed after RPT and cannot reasonably be considered a routine practice; however, biopsy samples should be regarded as valuable, as should liquid biopsy samples. Basal MHC-I expression could be increased using cytokines. IFN-γ, a cytokine therapy approved by the U.S. Food and Drug Administration, induces the expression of several genes that are related to MHC-I antigen processing and presentation. In 2019, in a pilot study (NCT01957709), Zhang et al. (50) showed that in patients with synovial sarcoma and myxoid or round cell liposarcoma with a cold tumor microenvironment, MHC-I expression and T-cell infiltration were increased after IFN-γ treatment. A phase 1 clinical trial (NCT02948426) is assessing IFN-γ-1b (Actimmune; Amgen Inc.) for intraperitoneal administration in recurrent chemotherapy-resistant ovarian cancer (51). A phase 2 trial reported that in patients with recurrent, platinum-sensitive ovarian, fallopian tube, and primary peritoneal cancer, administration of granulocyte–macrophage colony-stimulating factor and recombinant IFN-γ1b (52), before and after carboplatin, produced a satisfactory response and a manageable hematologic profile. The authors suggested more investigations into the components of this cytokine regimen for ovarian cancer (52). The presence of immune cells in the tumor microenvironment affects the tumor response to IFN-γ and tumors are highly heterogeneous, so the effects of exposure to proinflammatory IFN-γ on both tumor and immune cells (53*,*^54^) should be thoroughly investigated with a view toward testing combination therapies that include IFN-γ and RPT.

## CONCLUSION

We identified MHC-I expression level as a biomarker of RPT response in tumors receiving low absorbed doses. In tumors receiving high absorbed doses, immune system activation might be hindered by RPT-induced T-cell depletion. We previously showed that above a certain threshold absorbed dose, the benefit of increasing the irradiation is not clear. Therefore, these results indicate that therapeutic schedules based on repeated injections of high activities need to be reconsidered.

## DISCLOSURE

Julie Constanzo is or has been supported by Fondation ARC pour la Recherche sur le Cancer (ARCPJA32020060002266), LABEX MabImprove, and INCa PRT-K (PRT-K22-043). Jean-Pierre Pouget is or has been supported by SIRIC Montpellier Cancer Grant INCa_Inserm_DGOS_12553, INCa PCSI (C20025FS), Fondation ARC pour la Recherche sur le Cancer (ARCPJA32020060002266), Ligue Nationale Contre le Cancer (EL 2024.LNCC/JPP), LABEX MabImprove, Occitanie Region, and Cancéropôle Grand Sud Ouest. Jean-Pierre Pouget is a founding member of AlphaKen and a member of the scientific advisory board of Precirix. The Jean-Pierre Pouget team has industrial partnerships with Precirix, Roche, Orano, and NH TherAguix. No other potential conflict of interest relevant to this article was reported.

## Notes

KEY POINTS **QUESTION:** What is the immune system role in α-RPT efficacy? **PERTINENT FINDINGS:** Antitumor immunity driven by MHC-I can counteract low lesion absorbed doses in targeted radionuclide therapy. **IMPLICATIONS FOR PATIENT CARE:** The radiation immune stimulatory effects are clinically relevant in the current era of cancer immunotherapy, rendering systemic responses in patients an attainable aim.
